# Predicting prognosis in patients with stroke treated with intravenous alteplase through the 24‐h trajectory of blood pressure changes

**DOI:** 10.1111/jch.14331

**Published:** 2021-08-04

**Authors:** Kaiting Fan, Jie Zhao, Hong Chang, Xiaojuan Wang, Hui Yao, Xiaoxia Yao, Xin Yang

**Affiliations:** ^1^ Department of Neurology Xuanwu Hospital Capital Medical University National Clinical Research Center for Geriatic Disease Beijing China

**Keywords:** acute ischemic stroke, blood pressure trajectory, follow‐up, intravenous thrombolysis, neurological dysfunction

## Abstract

Blood pressure (BP) monitored within 24 h from the beginning of intravenous thrombolysis (IVT) with alteplase, is one of the important factors affecting the prognosis of patients with acute ischemic stroke (AIS). This study aimed to explore longitudinal BP trajectory patterns and determine their association with stroke prognosis after thrombolysis. From November 2018 to September 2019, a total of 391 patients were enrolled consecutively during the study period, and 353 patients were ultimately analyzed. Five systolic (SBP) and four diastolic blood pressure (DBP) trajectory subgroups were identified. The regression analysis showed that when compared with the rapidly moderate stable group, the continuous fluctuation‐very high level SBP group (odds ratio [OR]: 2.743, 95% confidence interval [CI]: 1.008–7.467) was associated with early neurological deterioration (END). Both the rapid drop‐high level SBP (OR: 0.448, 95% CI: 0.219–0.919) and DBP groups (OR: 0.399, 95% CI: 0.219–0.727) were associated with early neurological improvement (ENI). Moreover, there was a U‐shaped correlation between the OR value of SBP trajectory group and favorable outcome (the modified Rankin Scale [mRS] score 0–2) at 3 months: the slow drop‐low level SBP group represent a well‐established unfavorable outcome risk factor (OR:5.239, 95% CI: 1.271–21.595), and extremely high SBP—the continuous fluctuation‐very high level SBP group, are equally associated with elevated unfavorable outcome risk (OR:3.797, 95% CI: 1.486–9.697). The continuous fluctuation‐very high level DBP group was statistically significant in mRS (OR: 3.387, CI: 1.185–9.683). The BP trajectory groups show varying clinical features and risk of neurological dysfunction. The findings may help identify potential candidates for clinical BP monitoring, control, and specialized care.

## 1 INTRODUCTION

The clinical significance of monitoring and managing blood pressure (BP) during intravenous thrombolytic (IVT) therapy for the prevention of adverse prognosis in patients with acute ischemic stroke (AIS) remains an area of active investigation. Several observational studies have found that approximately 75%–80% of patients have elevated BP in response to stroke episodes,^1^, ^2^ and a high BP during the thrombolysis period is a risk factor for poor clinical outcomes, such as poor rate of recanalization, intracerebral hemorrhage, and neurological dysfunction.^3^, ^4^, ^5^ Interventional studies that used antihypertensive drugs to maintain BP within 141–150 mmHg or 130–140 mmHg have not reached a consensus on whether this leads to an improved prognosis.^6^, ^7^ In order to determine the ideal therapy, it is still necessary to accurately describe BP variations and their relationship with prognosis in patients undergoing venous thrombolysis.

In comparison, much less is known about the importance of changes in BP over time (ie, BP trajectories) in patients with AIS. A study on this topic showed that different BP trends were associated with the occurrence of cerebrovascular events.^8^, ^9^ Unsupervised functional principal components analysis was used to characterize SBP trajectories in patients with spontaneous intracerebral hemorrhage over first 24 h and their relationship to the unfavorable shift on modified Rankin scale (mRS).^10^ The method used to analysis BP trajectory has gradually received researchers’ attention and has a broad research prospect. For BP monitoring during IVT, current guidelines only indicate that BP should be maintained at < 180/105 mmHg within 24 h of treatment onset, mainly to prevent serious complications, such as symptomatic intracranial hemorrhage.^11^ Even if the ischemic penumbra is not affected by changes in infarction or massive hemorrhage, a varying BP trajectory causes unstable cerebral blood flow and cerebral perfusion pressure fluctuations, which will also affect the long‐term prognosis. Moreover, it is unreasonable to use a single BP value, the average BP, or BP variation‐related indicators at a single time point to represent the BP status within that time period. Studies describing the relationship between BP trajectories and early stroke outcomes in patients treated with IVT are lacking.

**WHAT IS ALREADY KNOWN ABOUT THE TOPIC?**

- Stroke guidelines indicate that monitoring BP within 24 h from the beginning of IVT in patients with AIS is essential due to the high incidence of complications.
- Several studies have shown that high SBP or DBP levels are associated with the prognosis of patients with AIS, such as changes in neurological function, hemorrhaging‐related complications, and mRS scores.
- In most similar studies, the BP values at a single time point, its mean value, the variation coefficient, and other indicators at multiple time points were used to describe BP and to explore the correlation between BP and stroke prognosis.

**WHAT THIS PAPER ADDS**

- According to BP data obtained at multiple time points in patients with AIS treated using thrombolysis, the group‐based trajectory model can be used to assess patterns of BP fluctuations.
- SBP and DBP showed different patterns as the time to thrombolytic therapy increased.
- There were differences in clinical characteristics among patients with different patterns of BP changes as well as in the degree of correlation with stroke prognosis, which was one of the independent influencing factors.
- Compared with these parameters, the BP values at a single time point, its mean value, the variation coefficient, and other indicators at multiple time points, which were used to describe BP in similar articles, BP trajectories are equally important values for predicting stroke prognosis.

Hypertension is a heterogeneous condition in patients with AIS, and available data support the importance of both systolic (SBP) and diastolic (DBP) BP monitoring. We designed our analysis using an unsupervised cluster approach, group‐based trajectory modeling(GBTM) approach that may provide an alternative method for summarizing long‐term BP values accounting for the dynamic nature of BP over time, to group similar longitudinal BP response patterns. We then evaluated the associations of these clusters, or most commonly BP parameters in similar articles, with neurological function changes and status using a standard multivariate regression approach. Improved knowledge of BP trajectories is critical in understanding the role of BP as a risk factor for adverse outcomes.

## 2 METHODS

### 2.1 Study sample

We prospectively identified 353 consecutive patients between November 2018 and September 2019 who were diagnosed with AIS and subsequently treated with IVT using alteplase, a thrombolytic medication, within 4.5 h after symptom onset and were then followed up for 3 months at one comprehensive stroke center (Xuanwu Hospital, Capital Medical University, Beijing, China). All patients were evaluated according to the American Heart Association guidelines and 2018 American Stroke Association guidelines for enrollment and contraindications before thrombolytic therapy was administered. All enrolled patients received 10% of the total dose of alteplase (Boehringer Ingelheim Pharma GmbH, Ingelheim, Germany), which was calculated as 0.9 mg/kg (maximum 90 mg), as an intravenous bolus, with the remaining 90% given as an infusion over the course of 1 h. Inclusion criteria were age ≥18 years, being treated with intravenous alteplase according to clinical guidelines, and follow‐up magnetic resonance imaging (MRI) or computed tomography performed 24 h after intravenous alteplase thrombolysis.^10^ Exclusion criteria were receiving subsequent endovascular treatment combined with IVT, no definitive evidence of focal hyperintensities in clinically relevant areas on initial or follow‐up diffusion weighted imaging, absence of prognostic data, missing information in the data collection form, and adverse post‐discharge outcome caused by a non‐stroke event, such as a fall or a fracture.

### 2.2 Blood pressure measurement

BP measurements were made during the routine care of patients with AIS. We used an electrocardiogram monitor (Intelli Vue MP70; Phillips Healthcare, Franklin, TN) to measure BP with patients lying in the supine position. All BP recordings and measurement time points were collected and stored in the electronic health record systems of the participating center. BP was monitored every 15 min for 2 h from the start of alteplase therapy, then every 30 min for 6 h, and then every hour for 16 h. Patients with BP higher than 185/110 mmHg before thrombolysis therapy or higher than 180/105 mmHg within 24 h from the beginning of IVT were treated by intravenous pumping of urapidil or nicardipine using the same protocol. In similar articles, we have seen that the time points focus on the admission, immediate completion of thrombolysis, 24 h, daytime and nightime in 24 h, and the most commonly used parameters are average value (mean), maximum value (max), minimum value (min), range (maximum–minimum), standard deviation (SD), and successive coefficient of variation (SV).

### 2.3 Outcome measures

The primary functional outcome was the mRS score at 90 days after stroke onset. Three months after the occurrence of ischemic stroke, the neurological impairment caused by stroke can be basically terminated. Walking function is the necessary function basis for patients to carry out daily living activities, and the improvement of walking ability in stroke patients is positively and linearly correlated with the future quality of life. Since the mRS scale takes the walking ability as a clear scoring standard, which was used the mRS score > 2 as the demarcation value of disability, it is widely used in stroke‐related studies and is one of the gold standards for evaluating the independent living ability of stroke patients. A favorable outcome was defined as an mRS score of 0–2 points, whereas an unfavorable outcome was defined as an mRS score of 3–6 points.^12^

The secondary outcome events included early neurological deterioration (END) and improvement (ENI). END was defined as an increase in the National Institutes of Health stroke scale (NIHSS) score ≥4 points or as an increase ≥2 points in one sub‐item that occurred at 24 h following alteplase infusion; these criteria were previously used to define significant deterioration.^13^ ENI was defined as an improvement in the NIHSS score ≥8 points or as a 0 or 1 score at 24 h following alteplase infusion.^14^

### 2.4 Covariates

Medical records were retrospectively reviewed by a nurse who was blinded to patients' outcomes and the following information was retrieved: demographic data (sex, age), medical history (hypertension, diabetes, coronary heart disease [CHD], atrial fibrillation, hyperlipidemia, stroke, transient ischemic attack [TIA], antiplatelets before stroke, repetitive thrombolysis, and pre‐stroke mRS score), vascular risk factors defined by our research team (body mass index [BMI], NIHSS score at admission [≤3 points was defined as mild stroke],^15^ gastric tube and catheterization after thrombolysis, pneumonia, using intravenous antihypertensive drugs, TOAST classification^16^), laboratory values (18 parameters from routine blood, biochemical, and coagulation tests), and characteristics of the thrombolytic procedure (onset to treatment time [OTT], door‐to‐needle time [DNT]).

### 2.5 Ethics statement

The study protocol was approved by the Ethical Committee of Xuanwu Hospital and conformed to the principles outlined in the Declaration of Helsinki. Written informed consent was obtained from all patients.

### 2.6 Group‐based trajectory modeling

During the 24 h after thrombolysis, we adopted a group‐based trajectory modeling approach using the traj procedure in SAS (SAS Institute, Cary, NC) to identify superior BP trajectories.^17^ Given the continuous measurement of BP value that belongs to censored continuous data, we used censored normal (CNORM) that is one of the three types of distributions provided by GBMT for our analysis. In a trajectory model, several regression models are estimated simultaneously through maximization of a likelihood that combines the information from all models. Specifically, based on individuals’ BP patterns over time, the probability of belonging to each potential BP group is modeled as a simple multinomial logistic regression. Each latent trajectory can be characterized by a starting value of impairment level (intercept) and possibly by a polynomial function (linear, quadratic, cubic), thereby capturing the start level and the shape of the BP course, respectively. For trajectories selection, the choice of the number of groups and the shape of each group are most important considerations. In our analysis, we first fit 2–6 group trajectories with all groups set to a polynomial function and then determination according to several statistical criteria and model‐fit indices described in below: ① Akaike's Information Criterion(AIC), Bayesian Information Criterion (BIC) were compared between different models. Smaller values of AIC and BIC denote better fit models. ② A minimum sample size in each group set at 5.0%. ③ Estimated probability of group membership for each trajectory group. The average of the posterior probability of group membership for each group should be greater than 0.7.

### 2.7 Statistical analysis

Continuous data were reported as means ± standard deviation and were analyzed using one‐way ANOVA or Kruskal‐Wallis tests as appropriate. Categorical data were presented as frequency and percentages and were analyzed using the chi‐square test. Logistic regression analysis and area under the curve (AUC) were used to determine the association between neurological damage and different BP trajectory groups, previous BP parameters. The strengths of the associations were determined by estimating the odds ratios (OR) and their 95% confidence interval (CI). To detect changes in associations between outcome and main exposures, the following multivariate logistic models were constructed: model 1 = no covariates; model 2 = statistical demographic indicators; model 3 = model 2 + all statistical indicators (*p* < .1). All statistical tests were two‐sided, and statistical significance was considered at *p* < .05. Statistical analyses were performed using SPSS v.22.0 (SPSS Inc., Chicago, IL).

## 3 RESULTS

During the 11‐month study period, 353 out of 391 patients met the study criteria and were enrolled in the study. Among the 12 patients with missing data, 1 patient was discharged from the hospital with the main diagnosis other than ischemic stroke, and 25 patients were lost to follow‐up. Among the included patients, 257 (72.8%) were male, 96 (27.2%) were female, and the mean age was 62.49±11.79 years.

### 3.1 Trajectory groups based on systolic blood pressure

**FIGURE 1:**
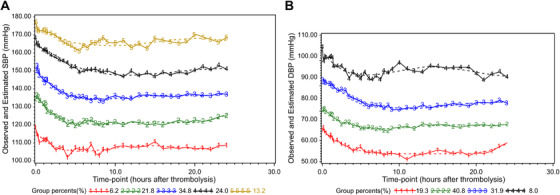
Trajectory groups of 24‐hour post‐thrombolysis blood pressure measurement. (A) Systolic blood pressure trajectories. (B) Diastolic blood pressure trajectories A. Systolic blood pressure trajectories; group 1 － slow drop‐low SBP group SBP=0.0007×t4‐0.038×t3+0.690×t2‐4.632×t+115.677 group 2 － rapid drop‐low SBP group SBP=0.0007×t4‐0.039×t3+0.749×t2‐5.822×t+136.173 group 3 － rapid drop‐medium SBP group SBP=0.0006×t4‐0.033×t3+0.701×t2‐5.849×t+151.043 group 4 － rapid drop‐high SBP group SBP=‐0.004×t3+0.232×t2‐3.639×t+165.549 group 5 － continuous fluctuation‐very high SBP group SBP=‐0.005×t3+0.223×t2‐2.773×t+174.543 B. Diastolic blood pressure trajectories. group 1 － rapid drop‐low DBP group DBP=0.0004×t4‐0.019×t3+0.379×t2‐3.199×t+72.943 group 2 － slow drop‐medium DBP group DBP=0.0003×t4‐0.015×t3+0.326×t2‐2.759×t+80.997 group 3 － rapid drop‐high DBP group DBP=‐0.003×t3+0.173×t2‐2.545×t+92.696 group 4 － continuous fluctuation‐very high DBP group DBP=0.0005×t4‐0.031×t3+0.601×t2‐4.175×t+103.607

Five groups were identified based on the SBP trajectory during the first 24 h after the beginning of IVT (Figure 1A). The groups describe the BP level and trend: group 1 (slow drop‐low SBP group, 102–114 mmHg, *n* = 22, 6.2%); group 2 (rapid drop‐low SBP group, 120–128 mmHg, *n* = 76, 21.8%); group 3 (rapid drop‐medium SBP group, 134–143 mmHg, *n* = 124, 34.8%); group 4 (rapid drop‐high SBP group, 150–157 mmHg, *n* = 84, 24.0%); and group 5 (continuous fluctuation‐very high SBP group, 162–173 mmHg, *n* = 47, 13.2%). In group 1, BP was relatively low with the highest point being below normal, and it fluctuated widely during the first 3 h. Groups 2, 3, and 4 showed stable BP after a rapid and steady decline within 1.5–2 h, whereas groups 2 and 3 had normal to high BP; the BP in group 4 remained stable at about 150 mmHg. In group 5, BP declined rapidly and steadily for the first 1.5 to 2 h, followed by wavy fluctuations.

**FIGURE 2:**
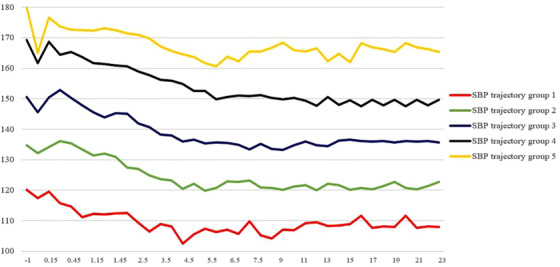
Original longitudinal SBP change. *Abbreviation*: SBP, systolic blood pressure.

**FIGURE 3:**
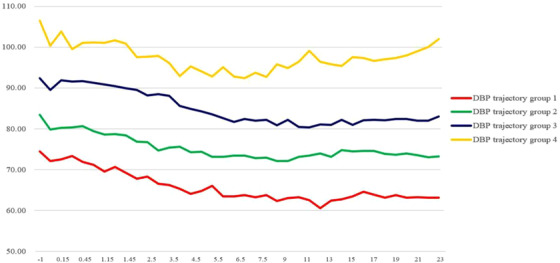
Original longitudinal DBP change *Abbreviation*: DBP, diastolic blood pressure.

Patients in these five SBP trajectory groups had distinct clinical profiles and laboratory results (Tables 1, 2). Patients in group 1 were significantly younger by about 10 years on average and had a lower prevalence of hypertension and diabetes by 23.4%–57.1% and 11.6%–30.7%, lower than the other groups, respectively. Patients with high BP were more likely to have chronic diseases; those in groups 4 and 5 had high BP and a high prevalence of both intravenous antihypertensive treatment and large artery atherosclerosis TOAST subtype. Blood glucose, glycosylated hemoglobin, and erythrocyte sedimentation ratio showed an increasing trend from group 1 to 5, whereas triglycerides and total cholesterol levels showed a “W” and “V” pattern, respectively. Both the international normalized ratio and prothrombin time decreased from group 1 to 5. The maximum or minimum values generally appeared in group 5.

**TABLE 1:** Patients characteristics by trajectory groups of 24 h blood pressure(n = 353)

	SBP trajectories						DBP trajectories				
Variables	1	2	3	4	5	*p* value	1	2	3	4	*p* value
Age	53.41±14.77	62.25±12.34	63.21±11.48	64.11±10.92	62.32±10.13	.004	63.28±10.33	62.12±12.56	61.79±11.23	65.17±13.20	.506
Male sex	14 (63.6%)	59 (77.6%)	89 (71.8%)	60 (71.4%)	35 (74.5%)	.728	51 (76.1%)	104 (71.7%)	80 (71.4%)	22 (75.9%)	.873
Hypertension	5 (22.7%)	35 (46.1%)	84 (67.7%)	67 (79.8%)	31 (66.0%)	<.001	41 (61.2%)	88 (60.7%)	73 (65.2%)	20 (69.0%)	.779
Diabetes	4 (18.2%)	25 (32.9%)	52 (41.9%)	25 (29.8%)	23 (48.9%)	.041	18 (26.9%)	55 (37.9%)	42 (37.5%)	14 (48.3%)	.205
Dyslipidemia	16 (72.7%)	62 (81.6%)	93 (75.0%)	68 (81.0%)	39 (83.0%)	.622	51 (76.1%)	113 (77.9%)	92 (82.1%)	22 (75.9%)	.741
CHD	2 (9.1%)	22 (28.9%)	24 (19.4%)	18 (21.4%)	9 (19.1%)	.285	8 (11.9%)	29 (20.0%)	28 (25.0%)	10 (34.5%)	.055
Atrial fibrillation	1 (4.5%)	10 (13.2%)	11 (8.9%)	9 (10.7%)	3 (6.4%)	.644	9 (13.4%)	13 (9.0%)	9 (8.0%)	3 (10.3%)	.675
Stroke	4 (18.2%)	23 (30.3%)	26 (21.0%)	17 (20.2%)	13 (27.7%)	.452	15 (22.4%)	32 (22.1%)	31 (27.7%)	5 (17.2%)	.587
TIA	0 (0)	3 (3.9%)	5 (4.0%)	1 (1.2%)	2 (4.3%)	.652	2 (3.0%)	5 (3.4%)	2 (1.8%)	2 (6.9%)	.454
Pneumonia	0 (0)	2 (2.6%)	3 (2.4%)	2 (2.4%)	2 (4.3%)	.887	3 (4.5%)	2 (1.4%)	1 (0.9%)	3 (10.3%)	.023
BMI (Kg/m^2)^	23.47±4.50	25.32±3.60	25.04±3.44	25.57±3.99	25.33±4.23	.222	24.75±4.02	25.21±3.59	25.40±3.99	25.05±3.62	.741
Antiplatelets therapy	3 (13.6%)	15 (19.7%)	20 (16.1%)	14 (16.7%)	7 (14.9%)	.940	8 (11.9%)	26 (17.9%)	19 (17.0%)	6 (20.7%)	.663
Smoking	10 (45.5%)	39 (51.3%)	51 (41.1%)	37 (44.0%)	23 (48.9%)	.853	31 (46.3%)	63 (43.4%)	53 (47.3%)	13 (44.8%)	.513
Alcohol drinking	7 (31.8%)	33 (43.4%)	44 (35.5%)	30 (35.7%)	21 (44.7%)	.688	27 (40.3%)	54 (37.2%)	48 (42.9%)	6 (20.7%)	.066
repetitive thrombolysis	0 (0)	2 (2.6%)	5 (4.0%)	3 (3.6%)	0 (0)	.585	5 (7.5%)	4 (2.8%)	1 (0.9%)	0 (0)	.091
OTT (min)	163.66±73.70	146.57±63.00	158.60±64.60	157.11±59.34	161.73±64.45	.700	157.37±65.62	158.01±65.08	155.78±62.13	148.39±57.94	.756
DNT (min)	30.79±12.47	26.91±12.36	30.69±14.71	30.61±16.62	30.43±13.43	.350	29.07±15.19	30.70±15.53	28.83±12.74	31.10±13.51	.895
Minor stroke	8 (36.4%)	26 (34.2%)	49 (39.5%)	20 (23.8%)	16 (34.0%)	.228	33 (49.3%)	51 (35.2%)	27 (24.1%)	8 (27.6%)	.006
Past mRS scores (0∼2)	22 (100%)	76 (100)	123 (99.2%)	83 (98.8%)	47 (100)	1.000	67 (100.0%)	143 (98.6%)	112 (100.0%)	29 (100.0%)	.738
Gastric tube	0 (0)	3 (3.9%)	1 (0.8%)	3 (3.6%)	4 (8.5%)	.108	1 (1.5%)	3 (2.1%)	4 (3.6%)	3 (10.3%)	.124
Catheterization	3 (13.6%)	3 (3.9%)	2 (1.6%)	3 (3.6%)	2 (4.3%)	.103	2 (3.0%)	2 (1.4%)	6 (5.4%)	3 (10.3%)	.079
Antihypertensive therapy	0 (0)	2 (2.6%)	10 (8.1%)	21 (25.0%)	21 (44.7%)	<.001	9 (13.4%)	21 (14.5%)	21 (18.8%)	3 (10.3%)	.608

**TABLE 2:** Comparison of laboratory parameters after thrombolysis(*n* = 353)

	SBP trajectories						DBP trajectories				
variables	1	2	3	4	5	*p* value	1	2	3	4	*p* value
WBC^a^ (*10^9^/L)	7.53±2.21	7.44±2.15	7.95±2.52	8.13±2.61	7.78±2.02	.447	6.99±1.94	7.99±2.47	7.97±2.38	8.43±2.56	.007
RBC^b^ (*10^12^/L)	4.36±0.68	4.33±0.45	4.42±0.50	4.39±0.54	4.43±0.50	.833	4.36±0.43	4.44±0.50	4.37±0.54	4.35±0.60	.718
PLT^c^ (*10^9^/L)	202.55±56.27	214.91±58.31	217.17±50.76	212.38±65.03	208.49±47.70	.579	202.30±54.32	214.97±54.12	220.95±55.55	203.00±66.19	.076
Urea^d^ (mmol/L)	5.49±1.78	5.10±1.82	4.92±1.38	5.03±1.71	5.21±2.11	.698	5.00±1.36	5.06±1.69	4.92±1.46	5.75±2.78	.506
Glucose (mmol/L)	5.93±2.75	6.45±2.76	6.64±2.73	6.92±3.16	8.22±3.53	.004	6.23±2.59	6.66±2.96	7.18±3.04	7.75±3.69	.023
UA^e^ (mmol/L)	310.91±91.09	318.26±91.26	316.18±84.96	325.61±90.36	328.06±78.02	.744	323.73±82.77	323.80±86.61	313.77±83.53	317.97±110.15	.882
TG^f^ (mmol/L)	1.86±2.46	1.40±1.04	1.69±1.20	1.40±1.06	2.04±2.55	.070	1.44±0.96	1.61±1.56	1.62±1.50	2.02±2.12	.673
Cholesterol (mmol/L)	3.97±0.85	3.90±0.94	4.10±1.05	4.09±1.11	4.92±3.10	.039	4.08±0.92	4.06±1.06	4.06±1.02	5.20±3.89	.094
LDL‐C^g^ (mmol/L)	2.44±0.80	2.34±0.80	2.48±0.87	2.43±0.89	2.76±1.00	.259	2.46±0.79	2.46±0.85	2.38±0.84	2.92±1.21	.121
PTA^h^ (%)	86.55±17.06	90.95±18.33	93.35±14.06	91.12±14.70	95.51±13.95	.034	94.97±16.17	91.34±16.70	91.51±13.35	92.34±14.93	.780
INR^i^	1.12±0.14	1.13±0.39	1.08±0.21	1.08±0.15	1.04±0.11	.028	1.08±0.19	1.10±0.30	1.08±0.20	1.07±0.12	.939
PT^j^ (s)	14.28±1.36	14.00±3.74	13.86±3.61	13.97±1.58	13.46±1.07	.016	13.68±4.71	14.04±2.73	13.84±1.84	13.77±1.22	.676
FIB^l^ (g/l)	2.26±0.61	2.52±0.94	2.52±0.64	2.56±0.75	2.67±0.65	.137	2.62±0.70	2.44±0.69	2.55±0.79	2.70±0.83	.197
HbAlc^m^ (%)	5.72±0.62	6.65±1.93	6.77±1.98	6.40±1.58	7.01±1.64	.028	6.37±2.03	6.51±1.67	6.88±1.79	7.21±2.28	.149
HCY^n^ (mmol/L)	18.65±13.48	15.11±8.05	18.13±14.36	15.81±7.76	20.98±15.09	.287	17.08±11.96	17.33±12.09	17.41±12.27	19.68±12.85	.839
D‐dimers (mg/ml)	1.74±3.07	3.57±5.66	2.22±3.44	4.40±12.20	2.07±3.25	.139	3.60±12.08	3.05±4.75	2.60±4.39	3.89±5.12	.176
Hs‐CRP^o^ (mg/L)	2.73±4.18	6.02±11.05	4.24±6.19	5.85±8.87	3.66±4.57	.228	2.89±3.84	4.85±6.96	5.76±10.08	5.32±9.26	.092
ESR^p^ (%)	4.20±3.63	8.74±12.77	7.53±6.64	8.63±6.21	9.46±11.96	.036	7.61±6.57	6.74±5.15	8.95±10.71	12.96±15.62	.323

^a^ White blood cell count.

^b^ Red blood cell count.

^c^ Platelet count.

^d^ Ureanitrogen, urea.

^e^ Uric acid, UA.

^f^ Triglyceride.

^g^ Low‐densitylipoprotein.

^h^ Prothrombin time activity.

^i^ international normalized ratio.

^j^ Prothrombin time.

^l^ Fibrinogen.

^m^ Glycated hemoglobin.

^n^ Homocysteine.

^o^ Hypersensitive C‐reactive protein.

^p^ Erythrocyte sedimentation rate.

### 3.2 Trajectory groups based on diastolic blood pressure

Four groups were identified based on the DBP trajectory (Figure 1B). Group 1 (rapid drop‐low DBP group, 62–69 mmHg, *n* = 67, 19.3%); group 2 (slow drop‐medium DBP group, 72–78 mmHg, *n* = 145, 40.8%); group 3 (rapid drop‐high DBP group, 82–88 mmHg, *n* = 112, 31.9%); group 4 (continuous fluctuation‐very high DBP group, 92–101 mmHg, *n* = 29, 8.0%). The only difference between groups 1–3 was the DBP level reached after the BP dropped.

Patients in these four DBP trajectory groups had distinct clinical profiles and laboratory results (Tables 1, 2). Group 4 had a higher proportion of coronary heart disease, pneumonia, and catheter insertion; higher NIHSS score at admission; and a greater likelihood of disease changes. The prevalence of drinking and repetitive thrombolysis decreased linearly among the four groups. White blood cell count, blood glucose, and total cholesterol levels increased from group 1 to 4, whereas the platelet count showed an inverted “U” shape. High‐sensitivity C‐reactive protein levels increased linearly in groups 1 to 3, whereas the values in group 4 decreased slightly.

The trend of the graph in Figure 2 and 3, which are drawn by calculating the average 24‐hour blood pressure of each track group, is similar to the result after applying GBT grouping.

### 3.3 Trajectory groups and stroke outcomes

After IVT, there were 67 (19.0%) cases of END, 131 (37.1%) cases of ENI, and 242 (68.6%) patients with an mRS score 0–2 at the 3‐month follow‐up. To examine the association between BP trajectory groups and outcomes, the groups were included as independent variables in a logistic regression model, and the moderate stable BP trajectory groups (SBP: group 3; DBP: group 4) were considered as the control groups (Table 3). Group 5 had a significantly increased risk of END (OR: 2.743, CI: 1.008–7.467) and the group 4 pattern was inversely associated with ENI (OR: 0.448, CI: 0.219–0.919). It is worth noting that there was a U‐shaped correlation between SBP trajectories and the mRS score at 90 days (low SBP: [OR: 5.239, CI: 1.271–21.595]; fluctuating high SBP: [OR: 3.797, CI: 1.486–9.697]). The rapid drop‐high level DBP group was inversely associated with ENI (OR: 0.399, CI: 0.219‐0.727) and group 4 showed an association with unfavorable outcome (OR: 3.387, CI: 1.185–9.683).

**TABLE 3:** Results of the association between trajectory groups of 24 hours blood pressure and stroke outcomes

	Systolic blood pressure Trajectory groups					Diastolic blood pressure Trajectory groups			
Stroke outcomes	Group 1	Group 2	Group 3	Group 4	Group 5	Group 1	Group 2	Group 3	Group 4
Early neurological deterioration(within 24 h)									
Model 1	0.263 (0.033,2.075)	1.140 (0.527,2.467)	Reference	1.507 (0.738,3.079)	2.852 (1.312,6.200)	0.885 (0.397,1.973)	reference	1.449 (0.776,2.704)	1.921 (0.762,4.842)
Model 2	0.328 (0.041,2.646)	1.241 (0.564,2.731)	Reference	1.503 (0.725,3.113)	3.175 (1.429,7.051)	0.884 (0.392,1.994)	reference	1.490 (0.788,2.815)	1.795 (0.690,4.670)
Model 3	0.995 (0.106,9.331)	1.343 (0.518,3.483)	Reference	1.407 (0.594,3.334)	2.743 (1.008,7.467)	0.851 (0.294,2.466)	reference	1.243 (0.576,2.684)	1.289 (0.355,4.680)
Early neurological improvement (within 24 h)									
Model 1	0.841 (0.335,2.111)	1.152 (0.650,2.042)	Reference	0.379 (0.205,0.701)	0.288 (0.128,0.645)	0.693 (0.383‐1.254)	reference	0.446 (0.263‐0.755)	0.443 (0.184‐1.066)
Model 2	0.815 (0.316,2.098)	1.125 (0.633,1.999)	Reference	0.380 (0.205,0.703)	0.282 (0.125,0.634)	0.686 (0.378‐1.246)	reference	0.442 (0.260‐0.751)	0.443 (0.183‐1.071)
Model 3	0.548 (0.155,1.932)	0.694 (0.327,1.472)	Reference	0.448 (0.219,0.919)	0.550 (0.214,1.409)	0.612 (0.309,1.214)	reference	0.399 (0.219,0.727)	0.516 (0.181,1.474)
Modified Rankin scale score (3–6) (3 months)									
Model 1	0.963 (0.327,2.838)	1.251 (0.651,2.402)	Reference	2.228 (1.220,4.068)	2.883 (1.420,5.851)	0.924 (0.477–1.791)	Reference	1.452 (0.851–2.478)	3.345 (0.851–2.478)
Model 2	1.377 (0.448,4.235)	1.284 (0.659,2.502)	Reference	2.222 (1.203,4.102)	3.096 (1.503,6.380)	0.891 (0.455–1.744)	Reference	1.508 (0.874–2.605)	3.146 (1.361–7.273)
Model 3	5.239 (1.271,21.595)	1.969 (0.866,4.477)	Reference	2.030 (0.967,4.262)	3.797 (1.486,9.697)	1.473 (0.652,3.327)	Reference	1.253 (0.655,2.396)	3.387 (1.185,9.683)

*Note*: In addition to SBP trajectory, high glucose levels also increased the risk of END (OR: 1.193, 95% CI: 1.049‐1.356,*p* = .007).

and MRS (OR: 1.096, 95% CI: 1.005–1.195, *p* = .038), Intravenous use of antihypertensive drugs was also associated with ENI.

(OR: 0.429, 95% CI: 0.187–0.983, *p* = .046).

Except for the systolic blood pressure trajectory, the occurrence of all three stroke outcomes was related to blood glucose.

(END: [OR: 1.125, 95% CI: 1.036–1.222, *p* = .005])∖(ENI: [OR: 0.916, 95% CI: 0.842–0.996, *p* = .039])∖(mRS: [OR: 1.103, 95% CI: 1.009–1.205, *p* = .031]). Urinary catheter indwelling within 24 h of thrombolysis was also one of the independent influencing factors of MRS (OR: 8.869, 95%CI: 1.613–48.772, *p* = .012).

### 3.4 Previous BP parameters and stroke outcomes

Blood pressure variability and stroke outcome in acute stroke patients, whether in patients with internal carotid artery occlusion or endovascular thrombectomy, which had shown that maximum values, max–min, SD and SV of systolic or diastolic BP resulted significantly higher in patients with poor outcome compared to those with good outcome after adjusting for potential confounders.^18^, ^19^ More parameters used to describe BP showed an association with END than ENI or MRS scores, which is one of the more important predictors of the risk of END. Moreover, the OR value of SBP or DBP on admission or immediate completion of thrombolysis was shown to be one of the better predictors of END than BP trajectory. Secondly, regardless of any parameter used to describe BP within 24 h, the diagnostic value of END was low(0.5 < AUC < 0.7). In the case of ENI, only BP trajectory and 24‐h SBP related indicators showed statistically significant correlation with ENI (*p* < .05). At the same time, the diagnostic value of SBP trajectory is not inferior to other BP parameters. With regard to the 3‐month MRS score, only BP trajectory showed a strong correlation with it. Other BP parameters could not be used as an indicator to predict the stroke functional status after 3 months; however, the mean and maximum values at any time point were more valuable for its diagnosis (Tables 4, 5). In conclusion, BP trajectory, as one of the main indicators for predicting and diagnosing the stroke outcome, is of equal significance compared with previous BP parameters.

**TABLE 4:** Results of the association between previous BP parameters and stroke outcomes

	Early neurological deterioration			Early neurological improvement			Modified Rankin scale score (3–6)		
Variables	OR	95%CI	*p*	OR	95%CI	*p*	OR	95%CI	*p*
SBP on admission	5.018	1.183,21.282	.029	0.197	0.023,1.660	.135	4.016	0.912,17.676	.066
DBP on admission	4.691	1.106,19.886	.036	0.230	0.028,1.894	.172	3.605	0.825,15.760	.088
Immediate SBP after thrombolysis	4.851	1.137,20.695	.033	0.227	0.394,1.109	.168	3.700	0.855,16.003	.080
Immediate DBP after thrombolysis	4.918	1.158,20.885	.031	0.215	0.026,1.791	.155	4.035	0.901,18.063	.068
24h SBP									
mean	1.021	1.002,1.040	.033	0.979	0.962,0.996	.017	1.013	0.991,1.035	.247
max	1.021	1.002,1.041	.031	0.983	0.969,0.996	.013	1.010	0.993,1.027	.256
min	1.028	1.004,1.052	.021	0.977	0.959,0.995	.015	1.011	0.989,1.034	.338
range	1.003	0.978,1.027	.834	0.990	0.971,1.009	.314	1.006	0.984,1.027	.611
SD	1.077	1.003,1.157	.040	0.934	0.882,0.988	.017	1.043	0.972,1.119	.238
SV	0.998	0.686,1.452	.992	0.747	0.555,1.005	.054	1.205	0.871,1.667	.261
24h DBP									
mean	1.019	0.987,1.052	.255	0.971	0.936,1.006	.104	1.029	0.990,1.070	.149
max	1.017	0.986,1.048	.283	0.987	0.962,1.013	.313	1.020	0.993,1.048	.152
min	1.022	0.980,1.066	.315	0.980	0.950,1.012	.218	0.996	0.962,1.030	.794
range	1.004	0.975,1.034	.801	1.000	0.976,1.024	.987	1.021	0.994,1.047	.123
SD	1.051	0.915,1.206	.484	0.907	0.808,1.019	.101	1.101	0.972,1.248	.130
SV	1.036	0.694,1.546	.864	1.088	0.789,1.502	.606	1.226	0.867,1.733	.249
SBP in daytime									
mean	1.015	0.997,1.035	.108	0.986	0.966,1.005	.154	1.012	0.990,1.034	.299
max	1.021	1.001,1.040	.036	0.991	0.975,1.008	.295	1.014	0.995,1.033	.148
min	1.024	1.002,1.046	.032	0.985	0.967,1.004	.129	1.010	0.990,1.031	.333
range	1.004	0.979,1.029	.770	1.004	0.983,1.025	.727	1.008	0.986,1.031	.461
SD	1.005	0.920,1.097	.912	0.999	0.927,1.076	.974	1.055	0.976,1.140	.181
SV	1.002	0.920,1.092	.958	0.976	0.913,1.043	.477	1.017	0.945,1.094	.655
DBP in daytime									
mean	1.018	0.988,1.049	.239	0.969	0.937,1.002	.065	1.022	0.985,1.059	.247
max	0.998	0.965,1.032	.909	0.983	0.957,1.010	.221	1.012	0.983,1.042	.419
min	1.020	0.982,1.059	.313	0.980	0.952,1.010	.188	0.999	0.968,1.032	.966
range	0.977	0.938,1.018	.270	1.000	0.973,1.028	.983	1.013	0.983,1.043	.401
SD	0.949	0.826,1.089	.455	0.970	0.873,1.078	.575	1.075	0.963,1.200	.199
SV	0.993	0.906,1.090	.889	0.963	0.887,1.045	.368	1.052	0.971,1.140	.216
SBP in nightime									
mean	0.999	0.947,1.054	.976	0.896	0.759,1.058	.196	1.100	0.925,1.307	.280
max	1.073	0.883,1.304	.481	0.896	0.758,1.059	.198	1.099	0.925,1.305	.285
min	1.101	0.909,1.332	.326	0.888	0.753,1.047	.158	1.117	0.944,1.323	.198
range	1.122	0.932,1.352	.225	0.869	0.736,1.026	.098	1.115	0.942,1.320	.204
SD	1.128	0.938,1.357	.201	0.869	0.736,1.026	.097	1.117	0.945,1.321	.195
SV	1.130	0.939,1.361	.195	0.871	0.738,1.029	.104	1.112	0.940,1.316	.214
DBP in nightime									
mean	1.137	0.945,1.367	.173	0.880	0.745,1.039	.133	1.125	0.952,1.330	.166
max	1.119	0.928,1.349	.239	0.879	0.744,1.040	.133	1.113	0.940,1.319	.214
min	1.134	0.943,1.364	.180	0.871	0.736,1.029	.104	1.098	0.932,1.293	.264
range	1.132	0.940,1.362	.191	0.869	0.736,1.025	.096	1.121	0.948,1.326	.181
SD	1.132	0.940,1.362	.191	0.869	0.736,1.026	.098	1.120	0.947,1.325	.187
SV	1.130	0.938,1.360	.199	0.867	0.734,1.023	.092	1.125	0.951,1.332	.170

**TABLE 5:** AUC of the association between BP parameters and stroke outcomes

	early neurological deterioration			early neurological improvement			modified Rankin scale score (3‐6)		
variables	AUC	95%CI	*p*	AUC	95%CI	*p*	AUC	95%CI	*p*
SBP on admission	0.582	0.529,0.634	.039	0.612	0.559,0.663	<.001	0.593	0.540,0.645	.004
DBP on admission	0.612	0.559,0.663	.005	0.571	0.518,0.624	.022	0.654	0.601,0.703	<.001
Immediate SBP after thrombolysis	0.586	0.533,0.638	.038	0.586	0.532,0.638	.006	0.575	0.521,0.627	.019
Immediate DBP after thrombolysis	0.580	0.527,0.632	.051	0.567	0.513,0.619	.035	0.633	0.580,0.683	<.001
24h SBP									
mean	0.602	0.549,0.654	.010	0.623	0.570,0.674	<.001	0.608	0.554,0.659	.001
max	0.603	0.550,0.654	.007	0.636	0.584,0.686	<.001	0.592	0.538,0.643	.005
min	0.600	0.547,0.652	.013	0.614	0.561,0.665	<.001	0.577	0.524,0.629	.023
Range	0.536	0.483,0.589	.367	0.569	0.516,0.621	.028	0.559	0.505,0.611	.071
SD	0.603	0.550,0.654	.009	0.623	0.570,0.674	<.001	0.608	0.554,0.659	.001
SV	0.541	0.487,0.594	.301	0.571	0.517,0.623	.024	0.565	0.511,0.617	.051
24h DBP									
mean	0.588	0.534,0.639	.032	0.576	0.522,0.628	.015	0.620	0.567,0.670	<.001
max	0.583	0.529,0.635	.048	0.565	0.511,0.617	.035	0.605	0.552,0.657	.002
min	0.586	0.533,0.638	.039	0.565	0.512,0.618	.036	0.591	0.538,0.643	.005
range	0.528	0.474,0.581	.502	0.528	0.474,0.581	.376	0.563	0.509,0.615	.068
SD	0.588	0.535,0.640	.031	0.576	0.522,0.628	.014	0.621	0.568,0.672	<.001
SV	0.524	0.470,0.577	.564	0.505	0.451,0.558	.883	0.546	0.493,0.599	.178
SBP in daytime									
mean	0.586	0.533,0.638	.029	0.607	0.554,0.659	<.001	0.604	0.551,0.656	.002
max	0.591	0.538,0.643	.025	0.603	0.550,0.655	<.001	0.606	0.552,0.657	.001
min	0.610	0.557,0.661	.006	0.608	0.555,0.660	<.001	0.592	0.539,0.644	.005
range	0.518	0.464,0.571	.669	0.508	0.455,0.562	.792	0.552	0.498,0.605	.130
SD	0.509	0.455,0.562	.832	0.525	0.472,0.578	.427	0.563	0.510,0.616	.062
SV	0.511	0.458,0.564	.781	0.523	0.470,0.576	.468	0.551	0.497,0.603	.127
DBP in daytime									
mean	0.582	0.529,0.634	.048	0.573	0.520,0.626	.019	0.601	0.548,0.653	.002
max	0.564	0.510,0.616	.116	0.556	0.502,0.608	.074	0.586	0.533,0.638	.009
min	0.579	0.525,0.631	.058	0.570	0.517,0.623	.023	0.578	0.525,0.630	.018
range	0.506	0.452,0.559	.893	0.506	0.452,0.559	.854	0.525	0.471,0.578	.467
SD	0.507	0.453,0.560	.873	0.525	0.472,0.578	.428	0.545	0.492,0.598	.192
SV	0.525	0.472,0.578	.426	0.525	0.472,0.578	.426	0.543	0.489,0.596	.220
SBP in nightime									
mean	0.612	0.559,0.663	.004	0.626	0.573,0.677	<.001	0.597	0.543,0.648	.004
max	0.628	0.576, 0.679	.001	0.630	0.577,0.681	<.001	0.599	0.546,0.651	.003
min	0.576	0.523,0.628	.057	0.615	0.562,0.666	<.001	0.572	0.518,0.624	.033
range	0.622	0.569,0.673	.002	0.543	0.490,0.596	.172	0.565	0.512,0.617	.053
SD	0.624	0.571,0.675	.002	0.555	0.501,0.608	.082	0.566	0.512,0.618	.054
SV	0.592	0.539,0.644	.019	0.550	0.496,0.602	.114	0.571	0.518,0.624	.032
DBP in nightime									
mean	0.561	0.507,0.613	.132	0.556	0.503,0.609	.073	0.595	0.541,0.646	.004
max	0.583	0.529,0.635	.043	0.548	0.495,0.601	0.123	.586	0.533,0.638	.010
min	0.610	0.557,0.661	.005	0.531	0.478,0.584	.322	0.587	0.534,0.639	.007
range	0.524	0.470,0.577	.563	0.518	0.464,0.571	.573	0.526	0.473,0.580	.433
SD	0.536	0.482,0.589	.382	0.527	0.473,0.580	.404	0.536	0.483,0.589	.285
SV	0.537	0.483,0.590	.357	0.523	0.470,0.576	.458	0.522	0.469,0.576	.502
SBP trajectory	0.606	0.553,0.657	.007	0.624	0.571,0.674	<.001	0.596	0.543,0.648	.004
DBP trajectory	0.566	0.513,0.618	.092	0.566	0.513,0.618	.002	0.585	0.532,0.637	.010

## 4 DISCUSSION

Five SBP and four DBP trajectory subgroups were identified in the first 24 h after initiating IVT using the group‐based trajectory model. In these groups, BP was classified according to its level as low, medium, and high and according to its changes as slow decline, rapid decline, and persistent fluctuation. Each trajectory group had different clinical characteristics, which were correlated with END, ENI, and mRS scores at 3 months. The continuous fluctuation‐very high SBP/DBP groups had the highest risk of having adverse events within 3 months. More importantly, the mean BP in these groups was similar, but they differed in the post‐stroke prognosis.

The mechanisms for acute BP response after stroke differ. The sensitivity of vascular baroreceptors decreased, and the ischemic penumbra partially or completely lost its ability to regulate the reflex autonomic modulation. The improvement of perfusion pressure was directly related to systemic BP. In addition, Increased intracranial pressure, elevated concentrations of circulating plasma catecholamine and inflammatory cytokine, stress from critical illness and hospitalization, unrecognized or uncontrolled pre‐existing hypertension, the Cushing phenomenon, dehydration, pain or discomfort, nausea, and hypoxia are the potential pathogeneses or critical influences that contribute to the acute BP change in patients with AIS.^20^, ^21^ Then, persistent lower BP level, insufficient blood oxygen supply to brain tissue, increased mitochondrial permeability and overexpression of inducible aquaporin may aggravate cerebral edema. Persistent higher BP level, the pressure difference between the cerebrovascular and brain interstitium, and the cracks in the vessel wall increases risk of brain edema and bleeding. As one of the common and interventionable indicators, BP is one of the main indicators with strong operability and practicability in clinical nursing work. By monitoring a patient's BP and its fluctuations over time, known as BP variability, more and better information can be obtained for observation.

Each indicator described BP, either the traditional method or the group‐based trajectory modeling, has its own advantages and limitations when considering the availability of appropriate data, ease of analysis, association with longer‐term clinical outcomes. Of course, all reduce the dynamic and longitudinal nature of BP into measurements that can be assessed cross‐sectionally at a single time point, which can lead to missed opportunities to understand BP course and potentially even misleading conclusions under certain circumstances. Emerging approach, group‐based Trajectory Modeling, which is to longitudinally measure BP provide nuanced assessments that reveal unique insights into different BP changes at different time points over an individuals’ treatment. This method help meet the needs of the current scientific agenda for BP changes and reveal important opportunities for developing more tailored interventions that target the varied care challenges patients may face over the intravenous thrombolysis within 24 h. However, this method indicated that outcomes must be relatively complete, and trajectory grouping and course are not fixed because of basing on the best fit to the observed data, not actually an innate characteristic.

BP in SBP group 1 fluctuated only within 3 h of admission. Considering that these patients were younger and had fewer chronic diseases, we believe that they were more affected by psychological factors, such as nervousness, fear of disease progression, side effects, and drug‐related complications. SBP group 5 had a higher NIHSS score at admission, indicating a greater influence of disease status. Meanwhile, with the highest proportion of intravenous use of antihypertensive drugs, patients in group 5 showed BP levels > 185/110 mmHg during admission and thrombolysis, which is likely to result in unstable BP over the 24‐h period. Changes in other groups suggested that although the blood pressure was high at admission, it was safe to reduce it quickly and steadily to about 160 mmHg later and to subsequently stabilize it. Therefore, it is particularly important to monitor BP at multiple time points in the acute phase, and clinicians can more intuitively grasp the changes in patients' condition according to the trajectory map.

Our study both confirms and expands on the findings of previous BP studies. First, a high BP level was prevalent in patients with AIS. Harper and coworkers^1^ found that 69.3%–82% of patients had BP > 140/90 mmHg and less than 5% of patients had a BP < 120 mmHg in the acute stage. In our study, 72% of patients had a higher‐than‐normal BP, and 6.2% of patients had a BP < 120 mmHg. Second, most patients’ BP levels gradually declined and stabilized over time within the acute phase and were associated with neurological deficits. The decrease in BP is faster within the first 8 h and lasts up to 36 h. Gill and coworkers^22^ found that an SBP decrease of 10 mmHg was related to a decrease of 0.51 points in the NIHSS scores. Zhang and coworkers^23^ and Tsou and coworkers^24^ et showed that SBP ≥160 mmHg or an increase of 15 mmHg could predict the risk of neurological deterioration. Leonardi‐Bee and coworkers^25^ also found that the relationship between SBP and the 14‐day mortality rate and 6‐month mortality or disability rate was U‐shaped rather than linear. The study showed that the risk of adverse prognosis increased by 5% for every 1‐mmHg increase up to 90 mmHg. Third, current research on the association between BP and prognosis mainly focuses on data from single measurements, multiple measurements in a short time, or 24‐h ambulatory blood pressure measurements. Current BP reporting methods may not adequately reflect individuals’ accurate BP levels. Group‐based trajectory modeling considers BP variations over time and the heterogeneity within multiple BP measurements, thus providing an effective approach to describe the relationship between BP changes and stroke outcomes.^26^, ^27^

Our study had several limitations that should be acknowledged. First, the participants were recruited only from one hospital; our findings should be verified in other cohorts to determine generalizability to other ethnicities and populations with different backgrounds. Second, identifying stroke outcomes based on more objective indicators, such as imaging findings, is more accurate. Third, due to the observational study design, although BP management was carried out according to guidelines, how long and how much the individual patients’ BP was controlled was left at the discretion of primary stroke physicians; future studies should aim to standardize this management.

## 5 CONCLUSION

Trajectory analysis models showed that the 24‐h changes in BP in patients with AIS treated with alteplase can reflect the dynamic changes in BP over time. BP may effectively be grouped according to distinct trajectory patterns, which have differential clinical characteristics and risk of subsequent early neurological improvement or deterioration as well as different associations with mRS score at 3 months. Being classified into the continuous low SBP (102–114 mmHg), fluctuating high SBP/DBP (162–173/92–101 mmHg), or rapidly high stable SBP/DBP (150–157/82–88 mmHg) groups was an independent predictor of adverse events. The clinical significance of this study is that our findings may help identify patients at a high risk of future vascular events and those requiring intervention.

## FUNDING

This study does not need any fund support, and all the work is completed within the normal work content and time. The data used in the study is also easy to access.

## CONFLICT OF INTEREST

There are no conflict of interest.

## AUTHOR CONTRIBUTIONS

**Conceptualization**: Kaiting Fan, Jie Zhao, Hong Chang, xin Yang.

**Data curation**:Kaiting Fan, Jie Zhao, Hong Chang.

**Investigation**:Kaiting Fan, Xiaojuan wang, Xiaoxia yao.

**Methodology**:Kaiting Fan, Jie Zhao.

**Project administration**:Kaiting Fan, Hong Chang, Hui Yao, xin Yang.

**Supervision**: Kaiting Fan, Hong Chang, Hui Yao. xin Yang.

**Visualization**:Kaiting Fan.

**Writing – original draft**: Kaiting Fan.

**Writing – review & editing**: Kaiting Fan, Jie Zhao, Hong Chang.

## References

[1] Harper G , Fotherby MD , Panayiotou BJ , Castleden CM , Potter JF . The changes in blood pressure after acute stroke: abolishing the ‘white coat effect’ with 24‐h ambulatory monitoring. J. Intern Med. 2010;235:343‐346. 10.1111/j.1365-2796.1994.tb01084.x 8151266

[2] Dawson J , Quinn TJ . Responses to acute stroke: beyond urgent imaging and systemic thrombolysis, where to now. J. R. Coll. Physicians Edinb. 2012;42:98‐100. 22693690 10.4997/JRCPE.2012.201

[3] Eryildiz ES , Özdemir AÖ . Factors Associated with Early Recovery after Intravenous Thrombolytic Therapy in Acute Ischemic Stroke. Noro Psikiyatr Ars. 2018;55(1):80‐83. 10.29399/npa.22664. PMID: 30042646; PMCID: PMC6045809. 30042646 PMC6045809

[4] Mattle HP , Kappeler L , Arnold M , et al. Blood pressure and vessel recanalization in the first hours after ischemic stroke. Stroke. 2005;36:264‐268. 15637309 10.1161/01.STR.0000153052.59113.89

[5] Delgado‐Mederos R , Ribo M , Rovira A , et al. Prognostic significance of blood pressure variability after thrombolysis in acute stroke. Neurology. 2008;71(8):552‐558. 10.1212/01.wnl.0000318294.36223.69. Epub 2008 Jun 11. PMID: 18550860. 18550860

[6] Anderson CS , Huang Y , Lindley RI , et al. Intensive blood pressure reduction with intravenous thrombolysis therapy for acute ischaemic stroke (ENCHANTED): an international, randomised, open‐label, blinded‐endpoint, phase 3 trial. Lancet. 2019;393:877‐888. 30739745 10.1016/S0140-6736(19)30038-8

[7] Marzan AS , Hungerbühler HJ , Studer A , Baumgartner RW , Georgiadis D . Feasibility and safety of norepinephrine‐induced arterial hypertension in acute ischemic stroke. Neurology. 2004;62:1193‐1195. 15079024 10.1212/01.wnl.0000118303.45735.04

[8] Portegies ML , Mirza SS , Verlinden VJ , et al. Mid‐ to Late‐Life Trajectories of Blood Pressure and the Risk of Stroke: the Rotterdam Study. Hypertension. 2016;67:1126‐1132. 27160196 10.1161/HYPERTENSIONAHA.116.07098

[9] Kim BJ , Cho YJ , Hong KS , et al. Trajectory Groups of 24‐Hour Systolic Blood Pressure After Acute Ischemic Stroke and Recurrent Vascular Events. Stroke. 2018;49(8):1836‐1842. 10.1161/STROKEAHA.118.021117. PMID: 30012819. 30012819

[10] Divani AA , Liu X , Petersen A , et al. The Magnitude of Blood Pressure Reduction Predicts Poor In‐Hospital Outcome in Acute Intracerebral Hemorrhage. Neurocrit Care. 2020;33(2):389‐398. 32524527 10.1007/s12028-020-01016-z

[11] Powers WJ , Rabinstein AA , Ackerson T , et al. 2018 Guidelines for the Early Management of Patients With Acute Ischemic Stroke: a Guideline for Healthcare Professionals From the American Heart Association/American Stroke Association. Stroke. 2018;49:e46‐e110. 29367334 10.1161/STR.0000000000000158

[12] Broderick JP , Adeoye O , Elm J . Evolution of the Modified Rankin Scale and Its Use in Future Stroke Trials. Stroke. 2017;48:2007‐2012. 28626052 10.1161/STROKEAHA.117.017866 PMC5552200

[13] Wu C , Sun C , Wang L , et al. Low‐Dose Tirofiban Treatment Improves Neurological Deterioration Outcome After Intravenous Thrombolysis. Stroke. 2019;50:3481‐3487. 31570084 10.1161/STROKEAHA.119.026240

[14] Ong CT , Sung SF , Wu CS , et al. Early neurological improvement after intravenous tissue plasminogen activator infusion in patients with ischemic stroke aged 80 years or older. J Chin Med Assoc. 2014;77:179‐183. 24657175 10.1016/j.jcma.2014.02.002

[15] Wang Y , Zhao X , Liu X , et al. Guidelines for the diagnosis and treatment of high‐risk non‐disabling ischemic cerebrovascular events. Chin. J. Stroke. 2016;11:481‐491. 10.3969/j.issn.1673-5765.2016.06.011

[16] Adams HP Jr , Bendixen BH , Kappelle LJ , et al. Classification of subtype of acute ischemic stroke. Definitions for use in a multicenter clinical trial. TOAST trial of org 10172 in acute stroke treatment. Stroke. 1993;24:35‐41. 7678184 10.1161/01.str.24.1.35

[17] Nagin DS , Odgers CL . Group‐based trajectory modeling in clinical research. Annu Rev. Clin. Psychol. 2010;6:109‐138. 20192788 10.1146/annurev.clinpsy.121208.131413

[18] Buratti L , Cagnetti C , Balucani C , et al. Blood pressure variability and stroke outcome in patients with internal carotid artery occlusion. J. Neurol. Sci. 2014 Apr 15;339(1‐2):164‐168. 24582284 10.1016/j.jns.2014.02.007

[19] Bennett AE , Wilder MJ , McNally JS , et al. Increased blood pressure variability after endovascular thrombectomy for acute stroke is associated with worse clinical outcome. J. Neurointerv. Surg. 2018;10(9):823‐827. 29352059 10.1136/neurintsurg-2017-013473

[20] Salman IM . Major autonomic neuroregulatory pathways underlying short‐ and long‐term control of cardiovascular function. Curr. Hypertens. Rep. 2016;18:18. 26838031 10.1007/s11906-016-0625-x

[21] AlSibai A , Qureshi AI . Management of acute hypertensive response in patients with ischemic stroke. Neurohospitalist. 2016;6:122‐129. 27366297 10.1177/1941874416630029 PMC4906555

[22] Gill D , Cox T , Aravind A , et al. A Fall in Systolic Blood Pressure 24 Hours after Thrombolysis for Acute Ischemic Stroke Is Associated with Early Neurological Recovery. J. Stroke Cerebrovasc. Dis. 2016;25:1539‐1543. 27053029 10.1016/j.jstrokecerebrovasdis.2016.03.002

[23] Zhang YB , Su YY , He YB , Liu YF , Liu G , Fan LL . Early Neurological Deterioration after Recanalization Treatment in Patients with Acute Ischemic Stroke:a Retrospective Study. Chin Med J(Engl). 2018;131:137‐143. 29336360 10.4103/0366-6999.222343 PMC5776842

[24] Tsou YJ , Lan KP , Fan JS . Relationship Between Changes in Prehospital Blood Pressure and Early Neurological Deterioration in Spontaneous Intracerebral Hemorrhage. Adv. Emerg. Nurs. J. 2019;41:163‐171. 31033664 10.1097/TME.0000000000000239

[25] Leonardi‐Bee J , Bath PM , Phillips SJ , Sandercock PA , Collaborative Group IST . Blood Pressure and Clinical Outcomes in the International Stroke Trial. Stroke. 2002;33:1315‐1320. 11988609 10.1161/01.str.0000014509.11540.66

[26] Kim BJ , Cho YJ , Hong KS , et al. Trajectory Groups of 24‐Hour Systolic Blood Pressure After Acute Ischemic Stroke and Recurrent Vascular Events. Stroke. 2018;49:1836‐1842. 30012819 10.1161/STROKEAHA.118.021117

[27] Zheng W , Mu J , Chu C , et al. Association of Blood Pressure Trajectories in Early Life with Subclinical Renal Damage in Middle Age. J. Am. Soc. Nephrol. 2018;29:2835‐2846. 30420422 10.1681/ASN.2018030263 PMC6287870

